# Retracted: Intrathecal Pump Implantation in the Cisterna Magna for Treating Intractable Cancer Pain

**DOI:** 10.1155/2021/9814903

**Published:** 2021-08-05

**Authors:** Case Reports in Anesthesiology

*Case Reports in Anesthesiology* has retracted the article titled “Intrathecal Pump Implantation in the Cisterna Magna for Treating Intractable Cancer Pain” [1] due to concerns with the reliability of the data. The authors identified that incorrect images were used in Figure 1 due to an error in the storage and image selection by the authors during manuscript preparation. The authors explained to the journal that the reliability of the results presented cannot be guaranteed, and the article is, therefore, being retracted with the agreement of the authors and the editorial board.
